# Characterization of Anthocyanins From Intraspecific Crosses of Monastrell With Other Premium Varieties

**DOI:** 10.3389/fnut.2021.664515

**Published:** 2021-04-16

**Authors:** R. Gil-Muñoz, J. D. Moreno-Olivares, D. F. Paladines-Quezada, J. A. Bleda-Sánchez, A. Cebrían-Pérez, M. J. Giménez-Bañón, J. I. Fernández-Fernández

**Affiliations:** Instituto Murciano de Desarrollo Agrario y Alimentario, Murcia, Spain

**Keywords:** intraspecific hybrids, Monastrell, grapes, wine, anthocyanins

## Abstract

One way in which the wine sector is reacting to the challenge of climate change is to develop plant material that is adapted to the new conditions. Such a strategy will allow the continuation of quality viticulture in traditional winemaking areas that will otherwise be abandoned. The objective of this study was to characterize the anthocyanin composition and content of selected intraspecific hybrids of Monastrell with two other varieties (Syrah and Cabernet Sauvignon). The experiment was carried out over three successive seasons, and the polyphenolic quality of the grapes and wines was assessed along with the adaptation of the hybrids to the high temperatures which will inevitably affect our area (south-eastern Spain). The results showed that, compared with grapes of the Monastrell variety and the wines made from them, most of the hybrids (MS10, MS34, and MC111) had a higher total anthocyanin concentration and overall content of acylated anthocyanins, depending on the year studied.

## Introduction

Grapevine is one of the most important fruit crops worldwide and constitutes a socio-economic activity of great significance for wine-producing countries (1). Spain has the largest surface area dedicated to grapevines of all the countries in the world (2). The area dedicated to this crop represents 5.6% of the total cultivated land of the country, and 98% of the grapes are wine varieties. Among red varieties, Monastrell is the sixth in terms of cultivated area, although this has been declining in recent decades due to its gradual substitution by other premium varieties, such as Cabernet Sauvignon or Syrah. Moreover, despite being very well-adapted to the Mediterranean area, Monastrell cannot avoid the consequences of climate change.

One of fighting back in this new scenario is to develop breeding programmes that will hopefully produce new varieties better adapted to the new conditions. However, two things must be borne in mind in this respect: the need to develop new varieties that are more resilient to climate change while maintaining the principal features cultivars, and increasing consumer demand for wines whose production is respectful of the environment. At the present time, crossbreeding and bud mutation are still regarded as the best ways for developing new grape cultivars (3).

Many different breeding programs are being developed around the world, and the progenies obtained as a result of these programs are still under evaluation. Several new cultivars have been generated by IMIDA (Institute of Research and Development in Agriculture and Food, located in south-eastern Spain), among them, ten red vinification grape crosses (five of them in awaiting registration), which are still being characterized for their quality and yield. The ten crosses are well-adapted to hot, dry climatic conditions, which should mean that will be suited to the forthcoming climatic scenario of the Mediterranean area.

Current data suggest that climate change may adversely affect grape and wine quality by affecting color, an important indicator for evaluating the quality of red wine and one of the most influential factors when consumers all over the world choose a wine. As is well-known, the color of red wine is mainly determined by the composition and concentration of anthocyanins, which are responsible for the slight red to dark purple color of wine. When studying grapes for winemaking, it is not only the quantity of anthocyanins that is important but also their anthocyanidin profile. The hydroxylation pattern of the B-ring is one of the main structural features of flavonoids and is an important determinant of their coloration, stability and antioxidant capacity (4).

Many factors can influence the concentration and profile of anthocyanins in grapes, which is primarily decided by genetic factors, with some modifications due to climate. Previous studies have shown the effect of temperature on the anthocyanin content of grape berries [e.g., (5–7)], and, in general, high temperatures have a detrimental effect on the content, playing a direct and important role in their formation (8). However, the distribution and concentration of grape anthocyanins mainly depend on the cultivar, maturity, production area and fruit yield (9, 10). For this reason, any variation in the anthocyanin content and the relative proportion of different anthocyanins can produce different phenotypes for skin pigmentation, with technological and nutritional consequences (11). Therefore, the anthocyanin profile can be used as a chemotaxonomical criterion to establish differences between grape varieties.

To the best of our knowledge, there have been few reports on the individual anthocyanin composition of new Monastrell varieties, and only two reports (12, 13) in which other hybrids were characterized previously. Therefore, the goal of this study was to evaluate the anthocyanin composition and content of ten interspecific hybrids of Monastrell made with Syrah and Cabernet Sauvignon, to assess the polyphenolic quality of grapes and wines, and to provide a tool for winegrowers facing the consequences of climate change.

## Materials and Methods

### Plant Material

The present study was carried out over three consecutive seasons (2017–2019) in an experimental field in Bullas (Murcia, south-east Spain). *Vitis vinifera* L. cv Monastrell was planted in 1997, and the intraspecific crosses were planted between 2007 and 2012. The vegetal material was obtained from intra-varietal crosses between Monastrell and Syrah (S) and Cabernet Sauvignon (C). There are 20 plants of each of these new varieties that are grafted on R110 and the planting density was 3,200 vines ha^−1^ (2.50 m between rows and 1.25 m between vines). The training system was a bilateral cordon trellised to a three-wire vertical system and drip irrigation was applied. Grapes were harvested at optimum technological maturity. Subsequently, grapes were transported to the winery (Estación Enológica de Jumilla), where wines were elaborated in accordance with a traditional vinification protocol in 50-L steel tanks.

### Vinifications

All vinifications were made using 50 kg of grapes, which were destemmed, crushed and sulfited (8 g SO_2_/100 kg). Total acidity was corrected to 5.5 g/L with tartaric acid, and selected yeasts were added (Uvaferm VRB, Lallemand, 25 g/hL). All vinifications were conducted at 25 ± 1°C. Throughout the fermentation pomace contact period (10 days), the cap was punched down twice a day, and the temperature and must density were recorded. At the end of this period, wines were pressed at 1.5 bars in a 75 L tank membrane press. Free-run and press wines were combined and stored at room temperature. The analyses were carried out at the end of alcoholic fermentation in triplicate.

### Physical-Yield Parameters in Grapes at Harvest

The yield per vine, and berry weight were determined. Total soluble solids (°Brix) were analyzed using an Abbé-type refractrometer (Atago RX-5000) and titratable acidity and pH were measured using an automatic titrator (Metrohm, Herisau, Switzerland) with 0.1 N NaOH.

### Anthocyanin Extraction Procedure

Grapes were peeled with a scalpel, and the skins were stored at −20°C until analysis. Samples (2 g) were immersed in methanol (40 mL) in hermetically closed tubes and placed on a stirring plate at 150 r.p.m. and 25°C. After 4 h, the methanolic extracts were filtered (0.45 μm) and analyzed by high-performance liquid chromatography (HPLC) injecting wines samples directly into the chromatograph.

### Identification and Quantification of Anthocyanins in Grapes and Wines by HPLC

The HPLC analysis was performed on a Waters 2690 liquid chromatograph (Waters Corporation, Mildford, MA, USA), equipped with a Waters 996 diode array, and a Primisep B2 SIELC column (Technologies, IL EEUU), 25 × 0.4 cm, 5 μm particle size and a CORTECS® Shield RP18 column (Crawford Scientific, Strathaven, United Kindom), 150 × 0.46 mm, 2.7 μm particle size were used to analyse the grapes and wines, respectively. In both analyses type were used as solvents 4.5% formic acid solution (solvent A) and HPLC grade acetonitrile (solvent B) at a flow rate of 0.9 mL min^1^. The compounds were identified according to Gil-Muñoz et al. (14). Compounds were compared with the UV spectra recorded with the diode array detector and those reported in the literature. In addition, HPLC–MS analysis was made to confirm the identity of each peak using an LC-MSDTrap VL-01036 liquid chromatograph-ion trap mass detector (Agilent Technologies, Santa Clara, CA) equipped with electrospray ionization (ESI). The heated capillary and voltage were maintained at 350°C and 4 kV, respectively. Mass scans were measured from 100 to 800 m/z and acquired in the negative ionization mode Data were processed using a Data Analysis 2.1 LC/MSD Trap software (Agilent). Anthocyanins were quantified at 520 nm, using malvidin-3-monoglucoside chloride as external standard (Sigma-Aldrich, Spain) (Supplementary Figures 1, 2; Chromatograms of anthocyanins in grapes and wines and compounds analyzed).

### Statistical Analysis

All the analyses were performed with the statistical package Statgraphics 5.1. The data were analyzed using analysis of variance (ANOVA) and a two-way analysis of variance (MANOVA) procedures, and means were separated by Duncan's multiple range test in order to highlight similarities and/or differences within samples (grapes and wines). Finally, discriminant analysis was used to identify the most discriminant variables.

## Results and Discussion

### Physicochemical Parameters at Harvest

The physical data of the progenies and of the Monastrell parental berries are presented in Table 1. Several parameters were evaluated at harvest time: kg/vineyard, berry weight, °Brix, total acidity and pH. In all three seasons, Monastrell variety was harvested the latest and the rest of the crosses were harvested between mid-August and the beginning of September, depending on the year.

**Table 1:** Physicochemical and production characteristics of grapes at harvest in the three seasons (2017–2019).

	**Monastrell**	**MC111**	**MC18**	**MC4**	**MC80**	**MC85**	**MC94**	**MC98**	**MS10**	**MS104**	**MS34**
**Harvest date**											
2017	20 Sept	5 Sep.	21 Aug	20 Sep.	3 Sep.	5 Sep.	21 Aug.	5 Sep.	10 Aug.	3 Sep.	21 Aug.
2018	17 Sep	3 Sep.	3 Sep.	3 Sep.	3 Sep.	17 Sep.	3 Sep.	3 Sep.	27 Aug.	3 Sep.	27 Aug.
2019	25 Sep	4 Sep.	26 Aug.	4 Sep.	9 Sep.	9 Sep.	26 Aug.	4 Sep.	22 Aug.	28 Aug.	22 Aug.
**Kg/vine**											
2017	1.93	2.80	2.32	1.96	1.36	1.81	3.15	3.14	2.33	3.19	1.83
2018	2.86	3.03	2.16	1.85	1.17	1.63	2.47	1.73	2.00	2.47	1.48
2019	3.12	3.26	2.66	2.61	2.11	2.63	3.44	2.52	2.54	5.37	1.68
**100 berry weight**^**1**^											
2017	172.17	101.83	101.20	97.10	117.23	126.07	106.83	142.07	110.43	131.00	100.73
2018	133.62	66.03	89.18	86.70	111.40	94.40	118.86	120.07	97.47	122.02	110.43
2019	138.97	60.94	68.41	67.49	98.47	83.92	95.81	94.25	97.33	79.23	86.62
**°Brix**											
2017	23.75 d	24.05 d	23.85 d	20.00 a	22.25 c	22.10 b	24.50 e	23.80 d	22.35 bc	22.60 c	24.60 e
2018	22.35 b	24.65 e	25.42 f	21.35 a	23.85 d	23.80 d	26.20 h	24.75 e	25.80 g	23.30 c	27.40 i
2019	22.40 f	23.00 h	22.20 de	17.65 a	20.95 c	22.65 g	22.10 d	20.55 b	23.25 i	17.55 a	22.30 e
**Total acidity**^**2**^											
2017	3.27 a	5.28 e	4.84 d	3.21 a	4.50 c	5.85 f	5.25 e	4.99 d	6.00 g	3.75 b	4.50 c
2018	2.91 a	5.53 g	5.10 e	3.76 b	4.45 c	4.54 c	5.50 g	4.75 d	3.19 f	4.69 d	5.71 h
2019	3.70 a	4.31 e	5.33 i	4.28 e	3.77 a	4.03 c	4.85 g	3.83 b	4.54 f	4.15 d	5.03 h
**pH**											
2017	3.96 h	3.76 d	3.58 a	3.72 c	3.62 b	3.64 b	3.62 b	3.93 g	3.58 a	3.89 f	3.79 e
2018	3.94 f	3.78 b	3.67 a	3.77 b	3.81 c	3.77 b	377 b	3.98 g	3.68 a	3.90 e	3.84 d
2019	3.80 i	3.64 e	3.52 b	3.65 ef	3.69 g	3.46 a	3.46 a	3.83 j	3.70 h	3.66 fg	3.54 c

*MC, Cross between Monastrell and Cabernet Sauvignon; MS, Cross between Monastrell and Syrah*.

1 *Berry weight expressed in g*.

2 *Total acidity expressed as mg/L tartaric acid*.

*Different letters in the same row reflect significant differences according to Duncan's test (p < 0.05)*.

There were differences in terms of the yield obtained for each variety and among years. The parental (Monastrell) plants had their highest yield in 2019 and the lowest in 2017. With regards to the crosses, in general, hybrids attained the lowest production level in 2018 and the highest in 2019. The hybrids with the lowest production were MC80 and MS34, while those with the highest production were MC94 and MS104, the last one reaching an exceptionally high level (5.4 kg/vine) in 2019. This may have been because in 2019 the pruning system was changed to pruning at the two bud site which resulted in an increase in the yield of all plants, although the climatological conditions may also have affected the results -so 2018 was the year with the lowest temperatures, and 2019 the year with the maximum radiation (Table 2).

**Table 2:** Climatic conditions during three seasons (2017–2019).

	**Month**	**Rmean**	**Tmax (°C)**	**Tmean (°C)**	**Tmin (°C)**	**Rainfall (mm)**
2017	June	322.15	28.22	23.89	19.15	3.80
	July	307.93	28.34	25.16	18.39	0.90
	August	244.80	29.35	24.54	18.63	41.3
	September	227.53	24.01	20.62	17.45	4.70
	Mean	275.60	27.48	23.55	18.41	12.68
2018	June	307.91	24.46	21.25	15.53	24.8
	July	323.17	27.04	25.35	22.65	0.00
	August	256.59	28.92	25.07	21.48	5.80
	September	194.59	24.45	21.44	17.11	56.7
	Mean	270.57	26.22	23.28	19.19	21.83
2019	June	329.86	25.33	21.38	17.80	0.90
	July	303.27	31.57	25.50	22.80	1.40
	August	271.84	28.43	24.94	21.37	22.70
	September	188.76	24.34	20.71	15.82	147.70
	Mean	273.43	27.42	23.13	19.45	43.18

*Rmean, mean radiation; Tmax, maximum temperature; Tmean, mean temperature; Tmin, minimum temperature*.

Berry size provides information about the skin surface/berry volume ratio, which can influence the winemaking process. Monastrell cultivar had significantly higher berry weights in the three seasons than the grapes of all the progenies. Only two of the crosses, obtained higher values during the two first seasons: MC98 and MS104. In the last season, the hybrids with the highest berry weight were MC80 and MS10. These data reflect the findings of Ortega-Régules et al. (15), who found that Monastrell berry weight (214 g per 100 berries) was double that of other grape cultivars, including Cabernet Sauvignon (104 g per 100 berries).

Total acidity also varied among the different cultivars analyzed during the 3 years studied. The highest value obtained in 2017 was that of MS10, in 2018 that of MC85 and in 2019 of MC18. During the 3 years the lowest values were obtained by the parental (Monastrell) although MC4 had similar values in the first season. Also the pH values varied during these 3 years, although in general the highest values were found in Monastrell and the lowest in the MC18 variety. These values suggest that the crosses are the well-adapted the new climatic scenario. Lower acidity levels are also frequently correlated with higher grape pH, although the relationship is affected by potassium accumulation, which is dependent on temperature (16). It has been suggested that potassium enters berry cells in direct exchange for protons, thus affecting berry and must pH at a given total acidity level (17).

The average soluble solids content of the studied grapes ranged from 20.0 (MC4) to 24.6 (MS34) °Brix, in the first season; 21.3 (MC4) to 27.4 (MS34) in the second season and from 17.5 (MS104) to 23.0 (MC111) in the third season. In 2019, the grapes were harvested 1 week earlier than in 2018. As mentioned above, climatological conditions could have influenced the results obtained, and, in general, °Brix values were higher in 2018, when the highest maximum temperatures were reached in August and the lowest rainfall was recorded (Table 2). Also, the two-bud pruning system practiced in 2019 would have favored earlier physiological maturity of the grape, allowing earlier harvest.

### Total Anthocyanin Content of Grapes and Wines

**Figure 1:**
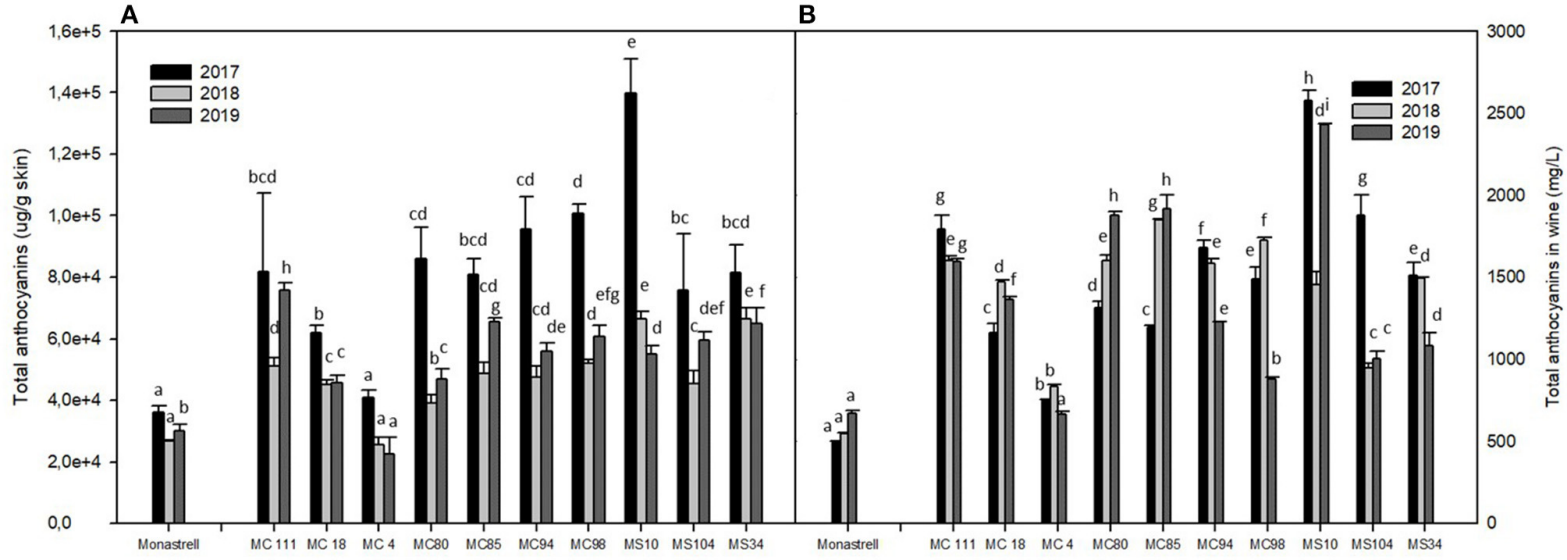
Total anthocyanins in grapes **(A)** and wines **(B)** in the three seasons (2017–2019).

The total anthocyanin concentrations found in grapes and wines from Monastrell and its hybrids are shown in Figures 1A,B, respectively.

The concentrations in grapes (expressed as μg/g skin) were higher in the hybrids than in Monastrell during the 3 years of the study, except in the case of the cross MC4 in 2018 and 2019. The appearance of a large number of hybrids in which the anthocyanin concentration is outside the range of concentrations found in their parental phenotypes is called transgressive segregation, and is a frequent occurrence in intraspecific crosses and in domesticated populations (12). Song et al. (18) evaluated the variability present in a segregating wine grape population derived from a cross between Graciano × Tempranillo and Hernández et al. (19) observed continuous variation and transgressive segregation in Grenache × Tempranillo population for all variables studied in the years evaluated. This indicates that genetic variability is present in the population studied.

As can be observed in Figure 1A, the total anthocyanin contents differed between cultivars and years, the highest concentrations being obtained in 2017. Climatic conditions are also important factors that affect anthocyanin synthesis in grape skin, and, related with this, all the progeny had a significantly higher anthocyanin content in 2017 than in 2018 and 2019 (Figure 1A). During the 2017 season, the contrast in temperatures between day and night was more pronounced, which would have contributed to a greater synthesis of anthocyanins. By contrast, in August 2018, the mean temperature was higher and rainfall lower than in 2017 and 2019 (Table 1). Besides this, torrential rainfall events was suffered in September of this year. Liang et al. (20) also found how climatic conditions had a strong influence on anthocyanin biosynthesis in hybrids from Muscat of Alexandría during the two seasons that these authors studied, while Bergqvist et al. (21) found that sunlight had a strong effect on anthocyanin concentration of Cabernet and Grenache grapes. Therefore, it is clear that climatic conditions such as high temperatures, more sunshine, lower relative humidity and rainfall is beneficial for anthocyanin synthesis in grapes.

As regards the hybrids, the highest concentration of total anthocyanins in 2017 was obtained in MS10, as it was in 2018, when it had similar values to MS34; in 2019 the highest levels were recorded for MC111, followed by MC85 and MS34. Of note is the fact that MS10 is one of the earlier varieties, since it is harvested at the end of August, when temperatures are highest (~35–40°C) and there is limited contrast between day and night temperatures. However, several authors, such as Shinomiya et al. (22) or Yamane et al. (6), have reported that anthocyanin accumulation is generally suppressed by high temperatures but promoted by low temperatures during ripening. Others authors, found that a high day and night temperature regime (37/32°C) completely inhibited the coloration of Emperor grapes (23) and that a high day temperature (35°C) strongly reduced anthocyanin concentration, although the effect of night temperature varied in relation with the day temperature considered (24). In light of the above, then, and based on our results, it would seem that MS10 is well-adapted to the current climatological conditions in our area.

As regards the wines at the end of alcoholic fermentation (Figure 1B), the lowest concentration of total anthocyanins in Monastrell wines was obtained in 2017 and the highest concentration in 2019. However, this high concentration did not coincide with a higher concentration of anthocyanins in the grape, probably because, genetically, the cell walls of Monastrell are known to have a more rigid structure, which would hinder anthocyanin extraction (13).

The concentration of total anthocyanins in the wines made with the hybrids varied with the year, and differed from the concentrations in grapes. This can be explained by the inherited properties of each of the crosses in terms of their cell walls, which may hinder or facilitate the extraction of anthocyanins during the wine-making process. However, other aspects also need to be taken into account; for example, Medina-Plaza et al. (25) reported that the phenol concentration in the final wine not only depends on the amount released from the grapes, but also on its interactions with the solids in the fermentor. Thus, in 2017, the highest concentration (2,579 mg/L) was obtained in the wines made with MS10, while in 2018 the wine made with the MC18 hybrid had the highest concentration (1,851 mg /L) and, once again in 2019, the wine made with the MS10 cross had the highest concentration, with a value of 2,429 mg/L. In some wines from certain crosses (MC111, MC94, MS10, MS104, and MS34), the concentrations were higher in 2017 than in 2018 and 2019. The same occurred in 2018 with the wines from the crosses MC18, MC4, MC98 and finally in 2019 in the wines from the crosses MC80 and MC85. Again, this may be due to year on year differences in the grapes at harvest or to the genetic material inherited by each hybrid, which would make them more similar to varieties such as Cabernet Sauvignon or Syrah, which have higher extractabilities than varieties such as Monastrell. Romero-Cascales et al. (26) studied the content and extractability of different varieties and found that, although Monastrell had the highest anthocyanin content, these compounds could be extracted more easily in wines from Cabernet Sauvignon, Merlot, and Syrah grapes.

### Profile of Anthocyanins in Grapes

The anthocyanin profile in grapes differed among the cultivars. They were present in non-acylated and acylated form as well as in combination with p-coumaric and caffeic acids. The category, the proportion and amount of anthocyanins in red grapes largely depend on the grape variety and the growing conditions, such as viticulture practices and the weather (27). It is generally accepted that the grape cultivar genotype essentially determines the anthocyanin composition, whereas environmental and cultivation conditions affect their accumulation in grapes by regulating the metabolism of polyphenols (28).

As can be observed in Table 3, the highest percentage of non-acylated anthocyanins in grapes was found in Monastrell in all of the seasons studied, followed by MC94, MC85, and MC111 hybrids. The percentages of the sum of non-acylated anthocyanins varied between 78 and 85% for Monastrell and 32 and 77% for the rest of the hybrids in the three seasons studied.

**Table 3:** Profile of anthocyanins in Monastrell grapes and their hybrids in the trhee seasons (2017–2019).

		**% Non-acylated**	**% Dp**	**% Cy**	**% Pt**	**% Pn**	**% Mv**	**% Acylated**	**% Acetates**	**% Coumarates**
2017	Monastrell	78.5 f	7.9 c	8.0 g	9.9 ef	12.6 h	40.0 f	21.5 a	4.0 a	17.4 d
	MC111	70.9 e	13.5 f	5.0 f	10.3 fg	7.9 f	34.0 b	29.3 b	16.6 c	12.3 c
	MC18	63.7 c	12.5 e	2.8 d	7.0 e	7.9 f	33.6 b	36.2 d	25.8 h	10.3 b
	MC4	37.2 a	2.8 a	0.2 a	3.5 a	0.8 a	29.8 a	62.8 f	23.7 g	38.1 f
	MC80	71.5 e	14.4 g	2.6 d	7.2 c	5.9 e	41.2 b	28.5 b	19.0 e	9.4 b
	MC85	71.9 e	17.8 h	2.6 d	10.6 g	2.7 b	38.2 e	27.9 a	18.9 e	9.4 b
	MC94	77.1 f	12.3 e	9.2 h	8.3 d	10.1 g	37.1 de	22.9 a	18.0 d	4.9 a
	MC98	66.3 d	14.1 fg	1.8 bc	10.0 ef	3.9 e	36.4 cd	33.7 c	20.1 f	13.4 c
	MS10	66.5 d	8.7 d	3.9 e	9.7 e	8.2 f	35.8 c	33.5 c	17.0 c	16.1 d
	MS104	55.8 b	4.9 b	1.2 b	9.9 b	5.0 d	39.8 f	44.2 e	19.7 f	24.2 e
	MS34	63.1 c	12.5 e	1.9 c	10.5 g	5.0 d	33.2 b	36.8 d	12.9 b	23.6 e
2018	Monastrell	81.4 h	6.4 b	6.8 f	9.2 de	9.2 de	47.4 d	18.5 a	3.3 a	15.1 e
	MC111	70.8 ef	14.1 g	5.0 cd	9.9 e	9.9 e	34.6 a	28.7 cd	16.2 bc	12.2 d
	MC18	69.2 e	12.8 g	5.4 de	7.5 b	7.5 b	36.4 a	30.8 d	22.1 e	8.6 bc
	MC4	49.7 a	4.6 a	1.2 a	4.7 a	4.7 a	37.1 ab	50.3 h	26.8 f	23.0 f
	MC80	72.1 f	10.6 d	6.4 ef	6.9 b	6.9 b	39.5 bc	27.7 c	19.5 de	8.1 b
	MC85	73.9 fg	13.4 fg	6.4 ef	9.2 de	9.2 de	36.8 c	26.1 bc	17.6 cd	8.5 bc
	MC94	76.2 g	12.9 f	8.8 g	7.9 bc	7.9 bc	35.8 a	23.9 b	19.8 de	4.1 a
	MC98	69.5 e	13.4 fg	4.2 cd	9.8 de	9.8 de	37.0 ab	30.5 d	19.7 de	10.6 cd
	MS10	66.7 d	8.8 c	3.9 c	9.8 de	9.8 de	35.9 a	33.3 e	16.9 bc	16.1 e
	MS104	56.8 b	4.8 a	0.8 a	5.4 a	5.4 a	40.2 c	43.3 g	21.0 e	22.1 f
	MS34	62.0 c	9.2 c	2.4 b	8.7 cd	5.7 b	35.8 a	38.0 f	14.7 b	22.8 f
2019	Monastrell	89.4 h	9.4 b	6.7 g	12.0 e	9.6 f	47.1 f	15.1 a	2.9 a	11.9 b
	MC111	69.5 f	15.0 d	3.7 de	11.6 e	6.4 e	32.8 ab	30.5 c	17.3 d	12.7 b
	MC18	62.5 cd	10.1 b	1.3 b	6.9 c	4.6 c	39.7 d	37.5 ef	26.1 h	11.1 b
	MC4	42.7 a	4.1 a	0.2 a	5.0 a	0.6 a	33.1 ab	56.9 h	26.5 h	29.0 e
	MC80	66.1 e	10.0 b	1.2 b	6.5 c	3.7 bc	44.7 e	33.9 d	22.0 g	11.5 b
	MC85	73.7 g	17.7 e	4.0 e	11.5 e	3.6 bc	40.3 ef	26.3 b	18.1 d	8.0 a
	MC94	74.0 g	14.8 d	5.9 f	9.8 d	6.2 e	37.3 bc	25.9 b	20.1 f	5.7 a
	MC98	64.7 de	11.8 d	1.5 b	9.8 d	3.0 b	38.6 cd	35.2 de	19.0 e	16.0 c
	MS10	65.3 de	10.5 b	3.2 d	11.5 e	6.1 e	34.0 b	34.6 de	18.0 d	16.4 c
	MS104	51.9 b	5.2 a	0.6 a	5.6 b	3.8 bc	36.8 c	48.1 g	16.4 c	31.1 e
	MS34	60.4 c	10.7 bc	2.4 c	10.1 d	5.1 d	32.1 a	39.7 f	13.9 b	22.7 d
**Multifactorial analysis**											
	Variety	^***^	^***^	^***^	^***^	^***^	^***^	^***^	^***^	^***^
	Season	^*^	ns	ns	ns	^*^	ns	^*^	ns	ns
	VxS	ns	ns	ns	ns	ns	ns	ns	ns	ns

*Dp, delphinidin; Cy, Cyanidin; Pt, Petunidin; Pn, Peonidin; Mv, Malvidin; MC, Cross between Monastrell and Cabernet Sauvignon; MS, Cross between Monastrell and Syrah; V, variety; S, Season; VxS, interaction between variety and season factors*.

For each parameter and season, different letters indicate significant differences between varieties according to Duncan test (p > 95%). Statistically significant a

* p < 0.05;

** p < 0.01 and

*** *p < 0.001; ns, not significant according to Duncant test*.

In general, hybrids had a higher percentage of tri-hydroxylate forms (delphinidin, petunidin and malvidin) than di-hydroxylate forms (cyanidin and malvidin), although Monastrell grapes had the highest percentage of cyanidin (6.7–8.0%) and peonidin (9.6–12.6%). Goméz-Plaza et al. (4) found that % cyanidin (Cy) represented 7–18% of the non-acylated anthocyanins in Monastrell and several other authors reported peonidin-3-glucoside as being the most prevalent pigment in the skins of the Spanish variety Garnacha Tintorera (29) and in a few Italian varieties such as Galliopo, Moscato Rosa and Nebbiolo (30). In this sense, the hybrid with the most similar values to Monastrell was MC94, which may be attributed to the lower activity of enzymes that control the formation of tri-hydroxylated anthocyanins in this variety. By contrast, the hybrid with the lowest percentage of di-hydroxylated anthocyanins was MC4. The anthocyanin composition of most of the progeny differed from that of their parents, and, in most cases, the profiles of the hybrids studied were closer to that of the other parental, in this case Cabernet Sauvignon or Syrah.

The different percentages of tri-hydroxylated monoglucosides obtained by the varieties studied are also shown in Table 3. The hydroxylation pattern of the B-ring is one of the main structural features of flavonoids and is an important determinant of their coloration, stability and antioxidant capacity and it is carried out by the enzymes flavonoid 3' hydroxylase and flavonoid 3'5' hydroxylase. Differences in the activity of these two enzymes will lead to differences in the di-hydrxylated/tri-hydroxylated anthocyanin ratio (4). As regards the tri-hydroxylated forms, our results showed that the percentage of delphinidin (Dp) was higher in all the hybrids than in Monastrell in all three seasons, except for MC4 and MS104 in 2017 and 2018, and in MC4 and MS10 in 2019. For every season the highest percentage of this compound was attained by a different hybrid: by MC85 in 2017 and 2019 and by MC111 in 2018 in. Roggero et al. (9) reported that the concentration of delphinidin-3-*O*-glucoside in grape berry increases during the early stages of berry ripening to reach a maximum value during this time, and subsequently decreases until harvest due to its conversion into petunidin-3-Oglucoside and malvidin-3-O-glucoside through the action of methyl transferases.

With respect to the percentage of petunidin (Pet) only in 2017, the following crosses provided higher percentages than Monastrell: MC111, MC85, and MS34. In a study made over two seasons (2015 and 2016), Gil-Muñoz et al. (13) also found that the percentage of this monoglucoside was higher in hybrids of Monastrell. Finally, the predominant anthocyanin in Monastrell and its hybrid cultivars was malvidin (Mv), which showed the highest percentage among the monoglucosides all 3 years: in Monastrell in 2018 and 2019, and the hybrid MC80 in 2017. Other authors, such as Goméz-Plaza et al. (4), also reported that malvidin-3-*O* glucoside had the highest percentage among the non-acylated anthocyanins in both Monastrell and Cabernet Sauvignon grapes, a finding echoed by Kyraleou et al. (31) in some Greek red varieties such as Agiorgitiko and Xinomavro. Indeed, malvidin-3-*O*-glucoside is the main anthocyanin in both grapes and wines of many European red vine varieties (32, 33). Carreño et al. (34) stated that the methylation capacity is higher in cultivars with a more intense pigmentation, which would explain why, in general, the pigmentation of the hybrids analyzed in this study was more intense.

Acylated anthocyanins are esters of the glucose moiety of free anthocyanins with acetic, p-coumaric or caffeic acids. In *Vitis vinifera* the presence of acylated anthocyanins is to be expected, but the quantity of this type of anthocyanin can vary among cultivars, as reported by several authors. For example, Ortega-Regules et al. (35) described a low level of acylated anthocyanins in Monastrell grapes from two different localities, while Mattivi et al. (30) mentioned they were lacking in Pinot Noir; lastly, Liang et al. (36) suggested that the nature of the acylated derivatives of anthocyanins in any variety will depend on climatic conditions, which, in turn, will depend on vineyard location.

The composition on acylated anthocyanins in Monastrell and their hybrids are shown in Table 3. Regarding the levels of acylates in grapes, all the hybrids showed a higher percentage throughout the study period than Monastrell variety (18%). Among the crosses, the percentage ranged between 29.3 and 62.8% in 2017, between 23.9 and 50.3% in 2018 and finally, between 25.9 and 56.9% in 2019. This low proportion of acylated anthocyanins in Monastrell has also been observed by other authors such as García-Beneytez et al. (29) and Gómez-Plaza et al. (4). Among the hybrids, it is worthy of note that the MC4 cross had the highest percentage of this type of anthocyanin in all three vintages with a value that ranged from 57 to 63%. The other crosses contained an average percentage of between 24% (mean of the 3 years) in MC94 hybrid and 45% (mean of the 3 years) in MS104 hybrid.

As regards acetates, the percentages shown by the hybrids were much higher than that seen in Monastrell, which had an average during the 3 years of 3% compared with the 26% for MC4, (close to that of Cabernet Sauvignon variety) and 14% for MS34. Zhang et al. (37) reported that Cabernet-Sauvignon and Syrah exhibited a high level of acetylated and coumarylated anthocyanins, respectively, so it is possible that the most of our crosses have a pattern of acylation closer to Cabernet Sauvignon or Syrah varieties.

Finally, coumaryl anthocyanin levels varied significantly among the cultivars and the values obtained were both higher and lower than those obtained by Monastrell, which showed a 3 year average of 15%. The hybrids that exceeded this percentage were the MC4 cross (30%, double that of Monastrell), MS104 (26%), MS34 (23%) and MS10 with 16% (profile more similar to Syrah). The values for the rest of the hybrids were lower values, ranging between 5% (MC94) and 13% (MC98). These results are important because coumaroylglucoside derivatives may also be more reactive and, consequently, more involved in the formation of derived pigments than other anthocyanins. Their higher reactivity has previously been observed during fermentation, when the rate of p-coumaroylvitisin formation was estimated to be higher than that of non-acylated vitisins (38).

Finally, Table 3 shows a multifactorial analysis performed by the different type of anthocyanins according to the variety, season and their interactions. The variety was the most dominant factor of variation for all types of anthocyanins (% non-acylated, % Dp, % Cy, % Pt, % Pn, % Mv, % acylated, % acetates, and % coumarates). Season was also the dominant factor in % non-acylated and % acylated. By contrast, no significant inter-annual differences were found among the anthocyanins.

The anthocyanin profile has been used as a chemotaxonomic parameter in the classification of red *Vitis vinifera* varieties (30, 39), and based on our results (Table 3) we conclude that anthocyanins could be considered useful markers for distinguishing among our grape varieties. However, although this characteristic should be used with care since anthocyanin content is heavily influenced not only by agronomical factors such as soil composition, irrigation, light intensity, but also by the climatic conditions of the year in question (40, 41).

### Profile of Anthocyanins in Wines

The results obtained for the non-acylated anthocyanins in wine are shown in Table 4. The total of non-acylated anthocyanins in the Monastrell wines and their hybrids were similar to those obtained in the grapes during the three vintages studied, the Monastrell wines showing the highest percentage (77.2–82.8%) followed by the MC94 and MC85 hybrid wines. Dimitrovska et al. (42) stated that anthocyanin profile of the grape berry skin and corresponding wine is similar and that there is a close correlation between the grape and wine anthocyanin patterns.

**Table 4:** Profile of anthocyanins in Monastrell wines and their hybrids in the three seasons (2017–2019).

		**% Non-acylated**	**% Dp**	**% Cy**	**% Pt**	**% Pn**	**% Mv**	**% Acylated**	**% Acetates**	**% Coumarates**
2017	Monastrell	77.2 h	5.6 b	3.1 g	9.9 j	8.8 j	49.7 h	20.7 a	9.1 a	10.3 c
	MC111	67.7 g	11.0 h	3.1 g	9.5 h	8.1 i	35.8 b	30.3 b	19.4 c	10.2 c
	MC18	61.2 c	8.2 c	2.1 e	6.1 b	6.3 h	38.5 c	37.1 g	25.1 k	11.5 d
	MC4	57.8 a	3.1 a	0.7 a	5.0 a	1.6 a	47.7 g	40.3 h	23.7 j	15.3 e
	MC80	66.9 f	9.1 e	2.0 de	6.4 c	5.4 e	44.0 f	31.2 c	20.4 e	10.4 c
	MC85	67.1 fg	10.1 f	1.9 d	8.5 f	2.8 b	43.8 f	30.7 bc	20.7 f	9.5 b
	MC94	67.7 g	10.3 g	3.5 h	7.7 e	6.4 h	39.8 d	30.6 b	21.4 g	8.6 a
	MC98	64.3 e	9.2 e	1.7 c	8.7 g	3.6 c	41.0 e	33.5 d	22.5 i	10.5 c
	MS10	64.6 e	10.5 g	2.7 f	11.9 k	5.8 f	33.5 a	34.3 e	21.8 h	12.0 d
	MS104	65.0 b	5.8 b	1.2 b	6.7 d	6.0 g	40.7 e	37.7 g	19.7 d	17.1 f
	MS34	63.4 d	8.7 d	1.1 b	9.8 i	4.5 d	39.3 d	34.9 f	19.1 b	15.0 e
2018	Monastrell	78.6 h	6.2 c	1.9 g	11.4 j	0.2 a	58.9 e	18.3 a	5.8 a	12.4 e
	MC111	66.9 ef	11.4 h	1.5 e	11.2 i	0.5 c	42.3 a	27.4 c	14.1 b	12.8 e
	MC18	64.0 c	11.4 h	1.6 f	8.0 d	0.4 c	42.5 a	31.0 e	19.3 e	11.4 bc
	MC4	61.7 b	5.0 b	0.3 a	6.4 b	0.2 a	49.8 d	34.9 f	20.6 f	14.0 f
	MC80	65.9 de	10.2 f	1.5 e	6.9 c	0.5 cd	46.8 c	29.1 d	16.9 d	12.8 cd
	MC85	69.4 g	12.9 i	1.4 d	10.1 fg	0.5 cd	44.4 b	25.7 b	14.7 b	10.7 ab
	MC94	67.3 f	10.9 g	3.0 h	8.4 e	0.6 cd	44.4 b	27.4 c	16.9 d	10.1 a
	MC98	64.1 c	11.0 g	1.0 c	10.1 f	0.5 cd	41.5 a	30.0 de	16.0 c	13.7 f
	MS10	64.1 c	7.0 d	1.4 d	10.4 gh	0.4 c	45.9 c	30.2 de	17.1 d	12.0 de
	MS104	57.2 a	4.1 a	0.7 b	5.9 a	0.3 b	46.2 c	37.1 g	21.3 f	15.1 g
	MS34	65.3 d	9.0 e	0.9 c	10.4 h	0.4 c	44.4 b	29.9 de	14.1 b	15.2 g
2019	Monastrell	82.8 e	7.6 c	1.9 g	11.2 i	7.3 f	54.8 f	15.7 a	5.1 a	10.6 b
	MC111	65.1 cd	12.3 h	1.2 e	10.8 h	5.1 e	35.6 a	30.1 bc	16.4 cd	13.2 de
	MC18	60.2 a	9.1 e	0.9 d	6.9 c	4.1 c	39.2 b	35.9 f	21.6 f	13.9 ef
	MC4	59.7 a	0.1 a	4.2 h	5.6 a	0.1 a	49.8 e	38.9 g	23.5 g	15.2 g
	MC80	61.7 ab	8.6 d	0.6 b	6.1 b	4.2 c	42.1 c	34.8 ef	19.7 e	14.9 fg
	MC85	67.0 d	13.8 i	1.3 e	10.0 e	3.2 b	38.7 b	28.8 b	16.9 cd	11.7 c
	MC94	67.5 d	11.3 g	1.9 g	8.3 e	4.6 c	41.4 c	28.7 b	19.2 e	9.4 a
	MC98	59.8 a	6.0 b	0.3 a	5.8 a	3.0 b	44.7 d	36.4 f	23.4 g	12.7 cd
	MS10	63.2 bc	9.6 f	1.5 f	11.1 i	4.7 d	36.3 a	33.5 de	15.9 c	17.3 h
	MS104	65.8 cd	0.1 a	5.2 i	6.7 c	4.8 de	49.1 e	32.1 cd	17.3 d	14.5 fg
	MS34	65.7 cd	9.6f	0.8 c	10.5 f	4.2 c	40.7 bc	31.0 c	14.5 b	16.3 h
**Multifactorial analysis**											
	Variety	^***^	^***^	ns	^***^	ns	^*^	^***^	^**^	^***^
	Season	ns	ns	ns	ns	ns	ns	ns	ns	^**^
	VxS	ns	ns	ns	ns	ns	ns	ns	ns	ns

*Dp, delphinidin; Cy, Cyanidin; Pt, Petunidin; Pn, Peonidin; Mv, Malvidin; MC, Cross between Monastrell and Cabernet Sauvignon; MS, Cross between Monastrell and Syrah; V, variety; S, Season; VxS, interaction between variety and season factors*.

For each parameter and season, different letters indicate significant differences between varieties according to Duncan test (*p* > 95%). Statistically significant a

* p < 0.05;

** p < 0.01 and

*** *p < 0.001; ns, not significant according to Duncant test*.

Regarding the percentage obtained for the different tri-hydroxylated and di-hydroxylated forms, the percentage of the former was always higher than that of the latter. It should be borne in mind that tri-hydroxylated anthocyanins (delphinidin, petunidin and malvidin-3-glucosides) are more stable in wines than di-hydroxylated ones (cyanidin and peonidin-3-glucosides) (43). The % Dp was highest in the MC111 wine in 2017 and in the wine made with MC85 in 2018 and 2019. The lowest percentages were obtained for the wine made from the MC4 and MS104 crosses. The % Pt during the 3 years studied was highest in MS10 wines in 2017 and in Monastrell in 2018 and 2019. Again, the wines from MC4, together with MS104 and MC98, provided the lowest percentages of this compound. The %Mv was higher than that of the other monoglycosides, as occurred in grapes. The highest values were obtained by Monastrell wines in all 3 years and the lowest values varied among the crosses with the season; thus, in 2017 the lowest % Mv was observed in the MS10 wines, in 2018 in the MC98, MC 111 and MC18 wines, and finally, in 2019, the MC111 and MS10 wines presented the lowest values.

Regarding acylated anthocyanins in wines, as can be observed in Table 4, the percentage of acylates in wines made with crosses was always higher than that obtained in wines made with the Monastrell variety. The presence of acylated anthocyanins is very important for a wine's color since they participate in intramolecular copigmentation processes, thus intensifying the color (44). On the other hand, acylated anthocyanins are more firmly entrapped in the matrix and form hydrogen bonds with polysaccharides, which can inhibit their extraction (45). The average obtained for acylated anthocyanins in the Monastrell wines over the 3 years was 20%, and while the average ranged between 32% for the wines made with the MC85 and MC94 crosses and 40% for the wine made with the MC4 cross.

As for the percentage of acetates and coumarates, as occurred in grapes, once again the percentage of acetates in the wines made with crosses was always much higher than that obtained by the wines made with the Monastrell variety, which had an average of 7% for the 3 years studied. The values obtained by the wines made with the hybrids were always two-fold, and in some cases three-fold or even four-fold the percentage obtained for the Monastrell wines. These values ranged from 16% for the wines made with MS34 to 23% for the wines made with MC4. Gil-Muñoz et al. (13) also found a higher concentration of acylated forms in wines from hybrids of Monastrell with varieties such as Cabernet Sauvignon and Syrah.

As regards the coumarates, as occurred with the grapes, both higher and lower percentages than those obtained in the Monastrell wines were found for the wines made with the crosses. The wines made with Monastrell grapes had an average of 11% coumarates in the three vintages studied. The wines made from the hybrids that had higher percentages than the Monastrell wines were MS10, MS104, and MS34, all of them with 15% coumarates, followed by the wine made with MC4 (11%) and the wines made with the crosses MC11, MC18, and MC98 (12% coumarates). Only the wines made with the MC85 and MC94 crosses had lower percentages than the wines made with the Monastrell variety.

Finally, Table 4 shows a multifactorial analysis performed by the different type of anthocyanins according to the variety, season and their interactions. The variety, just like it happened in the grapes, was the most dominant factor of variation for all types of anthocyanins except % Cy and % Pn. By contrast, season was not significant in any of the parameters analyzed. Many authors have reported the anthocyanin fingerprint of young wines obtained by classical fermentation can be considered a characteristic of each grape variety (46).

Finally, the interactions between the two factors (variety and year) were not statistically significant. This means that the anthocyanin profile, which differed among varieties, can be taken as an individual characteristic of each one, regardless of the interannual differences that may exist.

### Discriminant Analysis

A discriminant analysis was conducted using the following variables: % Dp, % Cy, % Pt; % Pn, % Mv, % acetates and % coumarates as independent variables to obtain the coefficients. The first two discriminant functions explained 82% of the variance. The first canonical function accounted for 60% of the variability and the second 30%.

**Figure 2:**
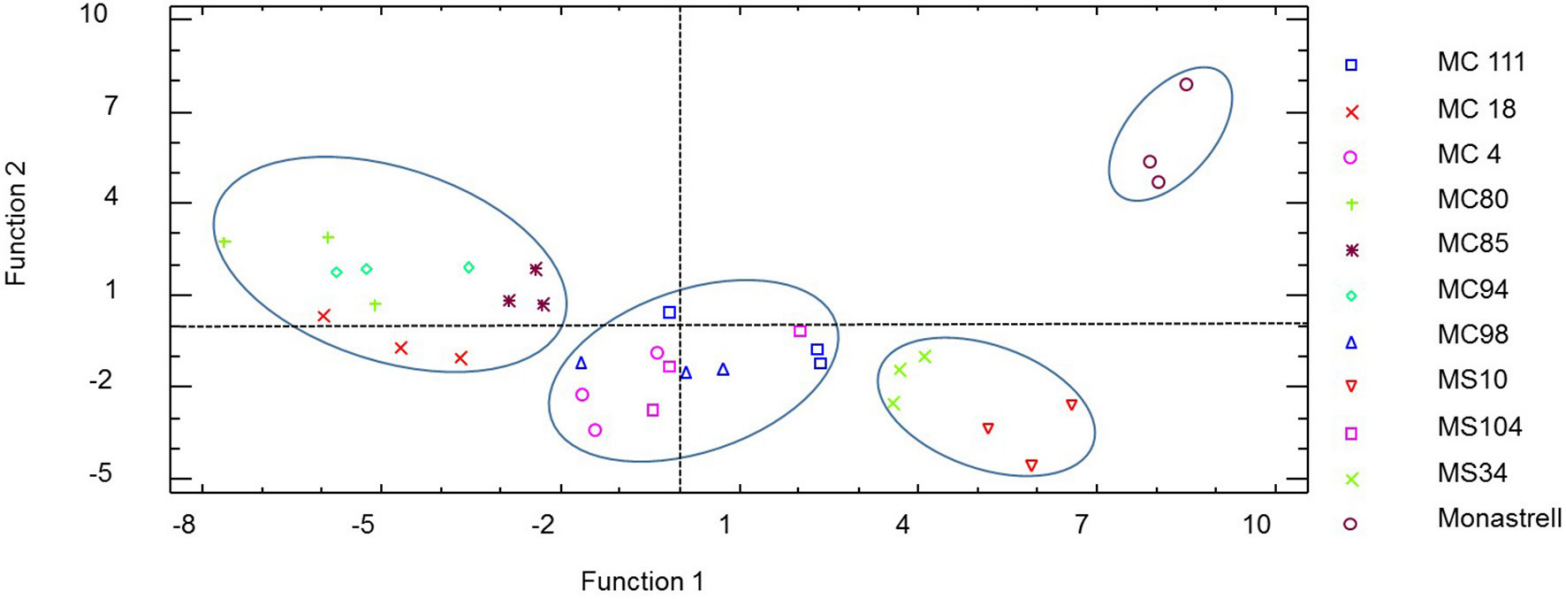
Distribution of varieties in the two-dimensional coordinated system by two first discriminant functions.

Figure 2 shows a lineal discriminant analysis of new varieties and the Monastrell as parental. The representation shows four different groups clearly separated according to the anthocyanin profile. As can be seen, the hybrids were distributed throughout the plot, MS10 and MS34 being closest to Monastrell and the hybrids MC94, MC85, MC80, and MC18 the most distant; surely due to corresponding to profiles of your other parentals (Cabernet Sauvignon or Syrah). The hybrids MS104, MC4, MC111, and MC98 lie in the middle of the plot.

The standardized coefficients of the discriminant functions (Supplementary Table 1) obtained showed that the variables with greatest influence on the first function were % delphinidin (in a negative way) and % petunidin (in a positive way); for the second function, the variables with the highest incidence were % acetates (in a negative way) and % malvidin and % delphinidin both (in a positive way).

Therefore, according to the groups of cultivars depicted in the plot, it may be said that the variables were able to discriminate varieties according to their anthocyanin profiles.

## Conclusions

The anthocyanin composition of 10 intraspecific red hybrids of *Vitis vinifera* from Monastrell variety growing in the southeast of Spain were analyzed.

In general, both the grapes and the wines of the new varieties showed a higher concentration of total anthocyanins, tri-hydroxylated forms and acylated compounds than the Monastrell variety. Based on the results obtained, it can be concluded that most of the hybrids selected have an anthocyanin profile far from that of Monastrell variety, and closer to that of the other parentals (Syrah or Cabernet Sauvignon). While there were interannual differences in terms of the concentration of the different types of anthocyanin, the profile was well-defined for each of these new varieties and their wines.

In summary, these results are indicative of the anthocyanin richness of the obtained hybrids compared with Monastrell and show their potential for producing quality red wines. This suggests that the ten selected hybrids may be useful for alleviating the effect of climate change, since, despite being harvested earlier than Monastrell, they contain higher concentrations of anthocyanins, suggesting they will adapt well to expected climatic conditions.

## Data Availability Statement

The original contributions presented in the study are included in the article/Supplementary Material, further inquiries can be directed to the corresponding author/s.

## Author Contributions

JM-O, MG-B, DP-Q, and AC-P: formal analysis. JM-O: data curation. JF-F: investigation. JF-F, RG-M, and MG-B: methodology. JB-S: conceptualization. RG-M: writing—original draft, funding acquisition, project administration, and resources. All authors: have read and agreed to the published version of the manuscript.

## Conflict of Interest

The authors declare that the research was conducted in the absence of any commercial or financial relationships that could be construed as a potential conflict of interest.
