# Supplementary material for: Characterization of Anthocyanins From Intraspecific Crosses of Monastrell With Other Premium Varieties
Source: Front Nutr. 2021 Apr 16;8:664515. doi: 10.3389/fnut.2021.664515 (PMC8085326; doi:10.3389/fnut.2021.664515)
Supplement: Supplementary file 1 [file Data_Sheet_1.pdf]

Figure 1A. Anthocyanins identified in grapes

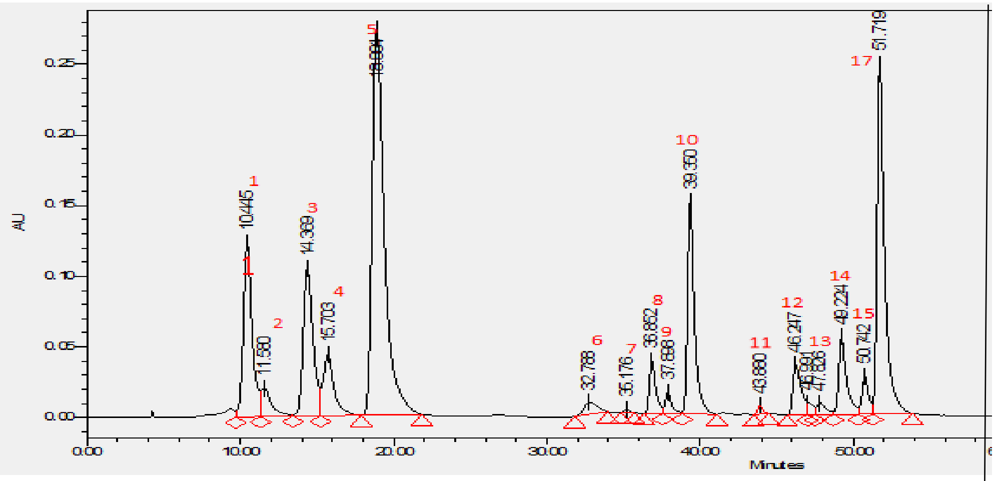

Compounds identified: (1) Delphinidin-3-glucoside; (2) Cyanidin-3-glucoside; (3) Petunidin-3-glucoside; (4) Peonidin-3-glucoside; (5) Malvidin-3-glucoside; (6) Delphinidin-3-acetyl glucoside; (7) Cyanidin-3-acetyl glucoside; (8) Petunidin-3-acetyl glucoside; (9) Peonidin-3-acetyl glucoside; (10) Malvidin-3-acetyl glucoside; (11) Malvidin-3-coumaroyl glucoside (cis); (12) Delphinidin-3-coumaroyl glucoside; (13) Cyanidin-3-coumaroyl glucoside; (14) Petunidin-3-coumaroyl glucoside; (15) Peonidin-3-coumaroyl glucoside; (16) Mv-3- coumaroyl glucoside (trans)

Figure 2. Anthocyanins identified in wines

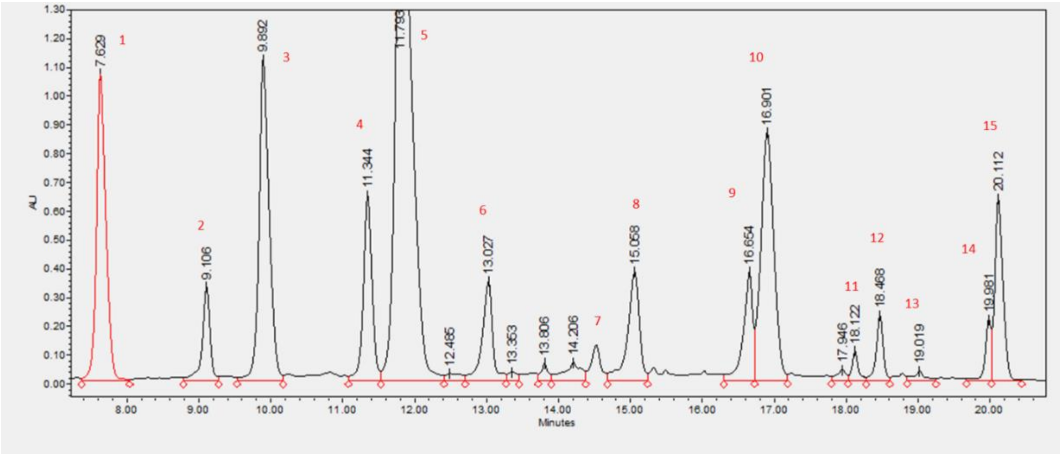

Compounds identified: (1) Delphinidin-3-glucoside; (2) Cyanidin-3-glucoside; (3) Petunidin-3-glucoside; (4) Peonidin-3-glucoside; (5) Malvidin-3-glucoside; (6) Delphinidin-3-acetyl glucoside; (7) Cyanidin-3-acetyl glucoside; (8) Petunidin-3-acetyl glucoside; (9) Peonidin-3-acetyl glucoside; (10) Malvidin-3-acetyl glucoside; (11) Malvidin-3-coumaroyl glucoside (cis);+Delphinidin-3-coumaroyl glucoside; (12) Cyanidin-3-coumaroyl glucoside; (13) Petunidin-3-coumaroyl glucoside; (14) Peonidin-3-coumaroyl glucoside; (15) Mv-3-coumaroyl glucoside (trans)
