# Supplementary material for: Characterization of Anthocyanins From Intraspecific Crosses of Monastrell With Other Premium Varieties
Source: Front Nutr. 2021 Apr 16;8:664515. doi: 10.3389/fnut.2021.664515 (PMC8085326; doi:10.3389/fnut.2021.664515)
Supplement: Supplementary file 2 [file Table_1.pdf]

Table 1. Discriminant function coefficients (standarized values) for varieties

	Function 1	Function 2
% Dp	-2.99	1.19
% Cy	-1.89	0.35
% Pet	2.29	-0.69
% Pn	0.49	1.36
% Mv	-0.99	1.46
% Acetates	-0.49	-0.71
% Coumarates	-0.76	-0.14
